# Oral Ethanol Treatment for Ethylene Glycol Intoxication

**DOI:** 10.7759/cureus.12268

**Published:** 2020-12-25

**Authors:** Misa Sasanami, Taihei Yamada, Takafumi Obara, Atsunori Nakao, Hiromichi Naito

**Affiliations:** 1 Center for Graduate Medical Education, Okayama University Hospital, Okayama, JPN; 2 Department of Emergency, Critical Care and Disaster Medicine, Okayama University Graduate School of Medicine, Dentistry and Pharmaceutical Sciences, Okayama, JPN

**Keywords:** ethylene glycol, oral ethanol treatment, ethylene glycol intoxication, throat pain

## Abstract

Ethylene glycol is an odorless, sweet-tasting liquid found in industrial solutions such as antifreeze and windshield wiper fluid. Brake fluid, an automobile transmission liquid, contains poisonous alcohols such as glycol ethers and ethylene glycols. The toxicity of ethylene glycol is associated with toxic metabolite production by the liver enzyme alcohol dehydrogenase. Administration of either intravenous ethanol or fomepizole, both of which competitively inhibit ethylene glycol metabolism by alcohol dehydrogenase and can prevent the production and accumulation of the toxic metabolites, can be used as an antidote. A 42-year-old male car mechanic was transferred to our hospital after accidentally ingesting approximately 100 mL of brake fluid. Immediately after ingestion, he threw up most of the ingested liquid; however, he complained of nausea and throat pain and was moved to our emergency department. The patient was successfully treated with administration of oral ethanol in the form of whisky through a nasogastric tube since neither intravenous ethanol nor fomepizole was available in our hospital at the time of his presentation. Our case demonstrates that oral ethanol can be used as an alternative treatment for patients with ethylene glycol intoxication.

## Introduction

Methanol and ethylene glycol poisonings are relatively common in emergency clinical practice [1]. These alcohols are relatively non-toxic by themselves; however, once they are initially metabolized by alcohol dehydrogenase, they can present with increased anion gap metabolic acidosis and elevated serum osmolality [2, 3]. Ethylene glycol intoxication, in particular, can lead to life-threatening complications, including metabolic acidosis and diverse secondary damage to the cardiopulmonary and renal systems if not properly treated [4]. Besides hemodialysis, effective therapy for ethylene glycol intoxication consists of administration of fomepizole and ethanol, both of which inhibit alcohol dehydrogenase in the initial step of the metabolism [5, 6].

We report successful treatment of a patient with ethylene glycol intoxication after accidentally ingesting brake fluid with the administration of oral ethanol in the form of whisky through a nasogastric tube. Fomepizole and intravenous ethanol have been traditionally used to compete for the active alcohol dehydrogenase site and decrease toxic metabolite formation [7]. However, as in our center, fomepizole or intravenous ethanol is not always available in some hospitals. Sharing our experience may help emergency physicians devise therapeutic strategies for similar cases.

## Case presentation

A 42-year-old male car mechanic was transferred to our hospital after accidentally ingesting approximately 100 mL of antifreeze. Immediately after ingestion, he threw up most of the ingested liquid; however, he complained of nausea and throat pain and was moved to our emergency department. On presentation, his vital signs were stable: Glasgow coma scale score 15/15, pulse rate 108 beats per minute, blood pressure 130/75 mmHg, and 36.7°C body temperature. Neurological tests, including examination of the cranial nerves, were unremarkable.

The patient reported no hematemesis, melena, or urinary symptoms. On abdominal examination, the patient had mild epigastric tenderness. Cardiovascular, respiratory, and neurological examinations were all unremarkable. Venous blood gas analysis revealed a pH of 7.363, partial pressure oxygen (PO₂) of 28.6 mmHg, partial pressure of carbon dioxide (PCO₂) of 49.1 mmHg, and base excess of -1.2 mmol/L. His serum osmolality was 286 mOsmol/L with an osmolar gap of 22.8 mOsm/kg and an anion gap of 11.4 mEq/L. No abnormalities like pleural effusion or consolidation appeared on the chest X-ray. His hemoglobin count was 13.8 g/dl, platelet count was 22.7 × 104/μL, and his white blood cell count was 107 × 102/μL. Serum creatinine and blood urea nitrogen were 0.91 mg/dl and 14.0 mg/dl, respectively. Results of a non-contrast computed tomography brain scan were normal.

As fomepizole was not available when the patient presented, oral ethanol therapy was started at 0.5 gram/kg, equating to a maintenance dose of 200 mL of whisky (25% alcohol) through a nasogastric tube every hour. The patient completely recovered without any complications and was discharged two days after the accidental ingestion.

## Discussion

Ethylene glycol is an odorless, sweet-tasting liquid used in industrial solutions such as antifreeze, windshield wiper fluid, brake fluid, and household industrial products because of its low cost [8]. While children sometimes accidentally consume ethylene glycol due to its sweet taste, adults tend to ingest it intentionally as an alcohol substitute or in a suicide attempt; it is used for homicidal purposes as well [9, 10]. The lethal ethylene glycol dose is reported to be 1.4 to 1.6 mL/kg [11].

Acute ethylene glycol intoxication progresses through three distinct stages: central nerve system depression (0.5-12 hours), followed by cardiopulmonary dysfunction (12-24 hours), and finally, renal dysfunction (24-72 hours) [2]. Ethylene glycol is quickly absorbed through the gastrointestinal tract, is oxidized to glycolaldehyde by alcohol dehydrogenase in the liver, and is then converted to oxalic acid, glyoxylic acid, and glycolic acid. Ethylene glycol toxicity is exerted via these metabolites directly or indirectly. Death usually occurs during the metabolic acidosis phase, which is associated with multiorgan failure and cerebral edema, the latter resulting in brain stem herniation and death [12]. During the metabolism of ethylene glycol, the changing point between the increased osmolal gap and anion gap greatly depends on the enzymatic activity of alcohol dehydrogenase, which is affected by body mass, exercise, nutrition, gender, age, and prior alcohol exposure [13]. Oxalic acid and calcium bind together, instigating the development of insoluble calcium oxalate crystals and occasionally bringing about hypocalcemia. The calcium oxalate crystals collect in various organs, leading to acute kidney failure and pulmonary, neurological, and myocardial dysfunction [4].

Treatment for these toxicities includes alcohol dehydrogenase inhibition, bicarbonate to reverse metabolic acidosis, and hemodialysis to enhance expulsion of the alcohols and their metabolites. Hemodialysis is considered a key element in managing severe ethylene glycol poisoning and is known to reduce hospital stay. Two antidotes are currently used to block alcohol dehydrogenase-mediated metabolism of ethylene glycol: fomepizole and ethanol, which minimize the formation of toxic metabolites [14]. Fomepizole is a potent alcohol dehydrogenase competitive inhibitor and has recently been used to treat metabolically-toxic alcohol poisonings. With an equivalent cost and greater safety associated with the pharmaceutical approach, fomepizole monotherapy has been recommended in minimally symptomatic patients. The administration regimen of fomepizole is easy, including a fixed loading dose followed by intermittent bolus doses every 12 hours, without need for fomepizole blood concentration monitoring [9]

Ethanol competes for alcohol dehydrogenase and has a greater affinity for the enzyme, making it useful for inhibiting the metabolism of ethylene glycol [15]. Although this is an appropriate treatment to prevent toxic metabolite production, ethanol therapy can be accompanied by substantial practical problems. Patients treated with ethanol should be closely monitored, as ethanol therapy can result in hypoglycemia, hepatotoxicity, and further central nerve system depression depending on the amount ingested and the patient’s sensitivity.

These adverse effects can confuse the interpretation of the clinical course or response to therapy [12]. To maintain the target ethanol level for adequate saturation of alcohol dehydrogenase to inhibit further metabolism of ethylene glycol to the toxic metabolites, a loading dose of 600-1000 mg/kg, followed by a maintenance dose of 1000-1500 mg/l, should be administered. During ethanol therapy, patients require intensive care, and close monitoring of blood concentration for ethanol and glucose is necessary every few hours [12].

Thus, oral ethanol administration for ethylene glycol intoxication may not be considered a universal guideline when fomepizole and hemodialysis are available. Recently, ethanol has largely been replaced by fomepizole as the antidote for toxic alcohols in many nations.

However, ethanol is still an important treatment option because the availability of fomepizole can be limited, its cost may be high, or the clinician may prefer it per his or her experience. We decided to administer oral ethanol in the form of whisky as an alternative treatment for our patient who had accidentally ingested ethylene glycol since fomepizole and hemodialysis were not readily available in our hospital. Further research into the most appropriate and cost-effective first-line treatment for ethylene glycol poisoning is required.

## Conclusions

Our experience and the previous literature demonstrate that oral ethanol administration can be a therapeutic option for ethylene glycol intoxication as a treatment for emergencies in hospitals where fomepizole is not readily available.
